# Hospital Length of Stay Prediction for Planned Admissions Using Observational Medical Outcomes Partnership Common Data Model: Retrospective Study

**DOI:** 10.2196/59260

**Published:** 2024-11-22

**Authors:** Haeun Lee, Seok Kim, Hui-Woun Moon, Ho-Young Lee, Kwangsoo Kim, Se Young Jung, Sooyoung Yoo

**Affiliations:** 1 Department of Biomedical Informatics and Data Science, Johns Hopkins School of Medicine Johns Hopkins University Baltimore, MD United States; 2 Office of eHealth Research and Businesses, Seoul National University Bundang Hospital Seongnam-si Republic of Korea; 3 Department of Transdisciplinary Medicine, Seoul National University Hospital Seoul Republic of Korea; 4 Department of Family Medicine, Seoul National University Bundang Hospital Seongnam-si Republic of Korea

**Keywords:** length of stay, machine learning, Observational Medical Outcomes Partnership Common Data Model, allocation of resources, reproducibility of results, hospital, admission, retrospective study, prediction model, electronic health record, EHR, South Korea, logistic regression, algorithm, Shapley Additive Explanation, health care, clinical informatics

## Abstract

**Background:**

Accurate hospital length of stay (LoS) prediction enables efficient resource management. Conventional LoS prediction models with limited covariates and nonstandardized data have limited reproducibility when applied to the general population.

**Objective:**

In this study, we developed and validated a machine learning (ML)–based LoS prediction model for planned admissions using the Observational Medical Outcomes Partnership Common Data Model (OMOP CDM).

**Methods:**

Retrospective patient-level prediction models used electronic health record (EHR) data converted to the OMOP CDM (version 5.3) from Seoul National University Bundang Hospital (SNUBH) in South Korea. The study included 137,437 hospital admission episodes between January 2016 and December 2020. Covariates from the patient, condition occurrence, medication, observation, measurement, procedure, and visit occurrence tables were included in the analysis. To perform feature selection, we applied Lasso regularization in the logistic regression. The primary outcome was an LoS of 7 days or longer, while the secondary outcome was an LoS of 3 days or longer. The prediction models were developed using 6 ML algorithms, with the training and test set split in a 7:3 ratio. The performance of each model was evaluated based on the area under the receiver operating characteristic curve (AUROC) and the area under the precision-recall curve (AUPRC). Shapley Additive Explanations (SHAP) analysis measured feature importance, while calibration plots assessed the reliability of the prediction models. External validation of the developed models occurred at an independent institution, the Seoul National University Hospital.

**Results:**

The final sample included 129,938 patient entry events in the planned admissions. The Extreme Gradient Boosting (XGB) model achieved the best performance in binary classification for predicting an LoS of 7 days or longer, with an AUROC of 0.891 (95% CI 0.887-0.894) and an AUPRC of 0.819 (95% CI 0.813-0.826) on the internal test set. The Light Gradient Boosting (LGB) model performed the best in the multiclassification for predicting an LoS of 3 days or more, with an AUROC of 0.901 (95% CI 0.898-0.904) and an AUPRC of 0.770 (95% CI 0.762-0.779). The most important features contributing to the models were the operation performed, frequency of previous outpatient visits, patient admission department, age, and day of admission. The RF model showed robust performance in the external validation set, achieving an AUROC of 0.804 (95% CI 0.802-0.807).

**Conclusions:**

The use of the OMOP CDM in predicting hospital LoS for planned admissions demonstrates promising predictive capabilities for stays of varying durations. It underscores the advantage of standardized data in achieving reproducible results. This approach should serve as a model for enhancing operational efficiency and patient care coordination across health care settings.

## Introduction

Length of stay (LoS) in the health care system directly impacts optimal patient care provision, health care costs, and overall hospital efficiency [1]. An accurate prediction of LoS allows health care institutions to optimize utilization, maximize the availability of limited hospital resources, and ensure the effective management of hospital personnel [2,3]. Precise predictions can also facilitate the identification of patients at risk for long-term hospitalization, facilitating timely interventions to improve treatment [4]. Consequently, LoS optimization is widely recognized as an essential strategy for efficiently managing hospital resources and the overall quality of health care services [5,6].

Thus far, however, hospital LoS prediction models have focused primarily on patients with specific diseases and binary outcomes within certain specialties, limiting both their applicability across broader hospital settings and the precision of day-specific LoS analysis [7-10]. Levin et al [11] developed an automated discharge prediction model for use in the multidisciplinary rounding process. Sotoodeh et al [12] proposed a framework based on a hidden Markov model to predict LoS for patients in an intensive care unit using physiological measurements. Deep learning and artificial neural networks, incorporating the entire electronic health record (EHR) and clinical free-text notes, have been utilized to predict LoS outcomes, specifically targeting medical-surgical patients [9]. The Bayesian network model also predicted an expected discharge within 7 days for emergency department (ED) admissions [13,14].

Furthermore, previous LoS prediction models, constrained by single-center nonstandardized data with limited covariates, reduced generalizability. Historically, models often included limited clinical features and overlooked aspects such as hospitalist services and the characteristics of the hospital stay, limiting the scope and accuracy of information in the developed models. LoS is reflected by multiple factors, such as environmental conditions, health care system dynamics, and social drivers of health [15,16]. In contrast, previous models for predicting LoS mostly centered on demographics, lab results, and vital signs. Traditional LoS models ignored admission details, procedure codes, medications, and comorbidities, which are crucial for understanding patient recovery [1]. Additionally, prior studies often relied on data from a single medical center and nonstandardized sources like administrative data, the Medical Information Mart for Intensive Care (MIMIC), and EHRs for model development, leading to models with limited transferability between institutions due to varied data standards [17-19].

This study used standardized observational clinical data to address these challenges. The Observational Medical Outcomes Partnership Common Data Model (OMOP CDM) is a standardized framework designed to streamline and harmonize health care data from diverse sources [20]. The OMOP CDM strengthens the semantic interoperability of data and enables large-scale studies, including various health care research applications like predictive modeling [21,22]. Specifically, it enhances the reproducibility of research findings and ensures comparability of results, allowing researchers to seamlessly work with data from various health care systems [23]. It also reduces the effort required for data mapping and transformation, thereby streamlining the process and improving the prediction model’s overall efficiency [22,23]. Adopting OMOP CDM in health care research contributes to more robust, scalable, and collaborative studies, ultimately improving the quality of health care research and patient care.

In this study, we aimed to develop and evaluate machine learning (ML)–based models for predicting hospital LoSs of 7 days or more for planned admissions using the OMOP CDM data. This study proposes multiclassification models for general hospitalization, targeting LoS durations of 3,4,5,6, and 7 days to reflect the complex clinical spectrum of health care conditions. These ML models are expected to facilitate an accurate prediction of LoS in various health care settings, aiding health care services in proactively implementing preventive measures to avoid unnecessary extensions of stay.

## Methods

### Data Source and Participants

**Figure 1:**
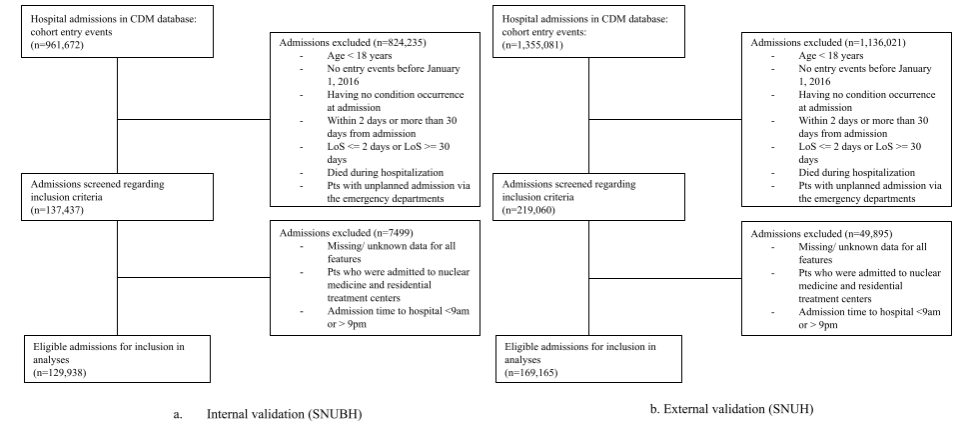
Flowchart for model development and external validation study populations. A. Internal validation was conducted at Seoul National University Bundang Hospital (SNUBH) B. External validation was conducted at Seoul National University Hospital (SNUH). CDM: Common Data Model; LoS: length of stay.

We carried out an observational cohort study using EHR data in the OMOP CDM format (version 5.3) from 2 tertiary hospitals, the Seoul National University Bundang Hospital (SNUBH) for development and internal validation and Seoul National University Hospital (SNUH) for external validation. SNUBH and SNUH are tertiary general hospitals affiliated with universities in the Seoul metropolitan area and the capital city, respectively. They provide services for inpatients, outpatients, and ED patients. The EHR data from both hospitals encompass patient demographics, diagnoses, chief complaints, outpatient drug prescriptions, inpatient and ED drug administrations, operations, vital signs, laboratory test results, and in-hospital deaths [24]. Both hospitals use the same EHR system and have similar ETL processes for OMOP CDM. Out of a total of 961,672 admission episodes, 137,437 (14.3%) indexed hospitalizations with LoS ranging from 2 to 30 days between January 2016 and December 2020 were included in the study. The prediction timing was set within 30 days, aligning with standard practices in most studies [25]. We included individuals who were over 18 years old, had at least 1 inpatient visit occurrence, were hospitalized in or after 2016, had at least 1 occurrence of any medical condition, survived hospitalization, and had no unplanned admissions during the study period. The study cohort of patients is provided in Figure 1.

### Ethical Considerations

Each participating site (SNUBH and SNUH) obtained an institutional review board (IRB) exemption (IRB no X-2103-672-907, IRB no E-2305-020-1429). Informed consent was waived due to the use of deidentified data within a secure internal network and the retrospective nature of the study.

### Data Extraction and Predictors

We used an open-source Feature Extraction R package (version 3.0.1; R Foundation for Statistical Computing) to extract patients’ clinical data before hospital admission from the OMOP CDM at SNUBH. Patient clinical characteristics, including condition occurrence, drug, observation, measurement, procedure, death, and visit occurrence tables were collected. The covariates included sex, age, the department at the visit, surgical operations, and lab results. After analyzing the LoS distribution for all patients, we excluded 2 departments, ophthalmology and nuclear medicine, from the model development due to their significantly shorter LoS (Figure S1 in Multimedia Appendix 1). Additional covariates that relied on the number of days (–365, –180, and –30) relative to the index date included diagnosis history, visit occurrences, and drug history. Severity scores such as the Charlson Comorbidity Index, Diabetes Complications Severity Index, CHADS2, and CHADS2-VAsc were also included as covariates. Additionally, custom covariates, such as the time, day of the week of admission, admission on holidays, day of the week of operation, whether operations were performed, diastolic and systolic blood pressure, body temperature, diagnosis at admission, and anthropometric measurements (eg, height and weight) were incorporated in the development of the prediction models.

### Outcomes

The study classified hospitalizations lasting 7 days or longer using binary classification. In comparison, multiclassification was applied to categorize hospital stays lasting 3 days or longer, with specific subgroups for 3, 4, 5, 6, and 7 days. A hospitalization was defined as an inpatient visit during the study period, excluding unplanned admissions such as hospitalization through the emergency room [26].

### Feature Selection and Model Development

After generating all the custom and noncustom covariates, logistic regression (LR) with Lasso regularization eliminated redundant collinear predictors from the models. We normalized continuous variables for uniform feature representation during data preprocessing and converted categorical variables into binary vectors using one-hot encoding. We removed variables from the analysis that had over 30% of the values missing [27]. Six ML models [5,28], including LR, random forest (RF), Extreme Gradient Boosting (XGB), Light Gradient Boosting (LGB), Gradient Boosting (GB), and multilayer perceptron (MLP) were evaluated to develop predictions for binary (LoS ≥7 days) and multiclass length of stay (LoS 3, 4, 5, 6 ≥7 days) outcomes based on the selected features. Model hyperparameters were optimized using 10-fold cross-validation via RandomizedSearchCV from Python (v.3.7.6) and scikit-learn library (v.0.22.1). We externally validated the models using SNUH data.

### Model Evaluation

We evaluated model performance using 5 metrics: area under the receiver operating characteristic curve (AUROC), area under the precision-recall curve (AUPRC), sensitivity, precision, and specificity. A bootstrap resampling technique with 1000 replicates was employed to compute 95% CIs for all metrics. The optimal threshold point of the Youden Index was used to assess the sensitivity and specificity of an LoS of 7 days or longer. Shapley Additive Explanations (SHAP) analysis interpreted the output of ML models and identified predictors that positively and negatively contribute to the model’s prediction. Furthermore, calibration plots assessed the reliability of the prediction models and ascertained whether the predicted probabilities aligned with the actual outcomes across different probabilities. The Brier Score measured the accuracy of probabilistic predictions quantitatively. The performance of the models at SNUH was evaluated using AUROC and AUPRC, SHAP, and calibration plots alongside Brier Scores.

### Statistical Analysis

Statistical analysis was conducted using R software (version 3.0.1) and Python (version 3.8.10).

Patient entry events consisted of 2 groups based on LoS <7 days and LoS ≥7 days. For the internal data set, comparisons between these groups were performed using standard 2-sample *t* tests for continuous variables and chi-squared tests of independence for categorical variables. To compare characteristics between internal and external validation cohorts, we used the *tableone* package in R, which generated descriptive statistics presenting continuous variables as mean (SD) and categorical variables as frequency counts and percentages. All the packages used are listed in Table S2 in Multimedia Appendix 1.

## Results

### Baseline Characteristics

During the study period, we identified 129,938 inpatient admission episodes at SNUBH and 169,165 at SNUH, each with an LoS ranging from 2 to 30 days. The mean age of the admitted patients were 58.4 (SD 15.4) years at SNUBH and 59.2 (SD 14.9) years at SNUH. The average LoS for planned admissions was 5.2 days at SNUBH and 5.4 days at SNUH. The baseline characteristics of both cohorts are presented in Table 1.

**Table 1:** Baseline characteristics and outcomes of planned admissions in the test and validation cohorts.

Characteristics			Internal validation set (SNUBH^a^)							External validation set (SNUH^b^)				
			LoS^c^< 7 (n=95,499)		LoS>=7 (n=34,439)		Overall (n=129,938)		LoS <7 (n=122,137)			LoS >=7 (n=47,028)		Overall (n=169,165)
Age (years), mean (SD)			57.5 (15.5)		60.9 (15)		58.4 (15.4)		59.4 (14.6)			58.8 (15.5)		59.2 (14.9)
Male sex, n (%)			42,117 (44.1)		16,762 (48.7)		58,879 (45.3)		63,956 (52.4)			21,986 (46.8)		85,942 (50.8)
Height (cm), mean (SD)			162 (8.75)		161 (9.08)		162 (8.84)		162 (8.70)			162 (8.86)		162 (8.75)
Weight (kg), mean (SD)			63.6 (12.7)		62.9 (12.4)		63.4 (12.6)		63.3 (12)			62.5 (12.3)		63 (12.1)
Body temperature, mean (SD)			36.7 (0.4)		36.8 (0.4)		36.7 (0.4)		36.4 (0.392)			36.5 (0.446)		36.5 (0.409)
**Care site, n (%)**														
	Allergy	213 (0.2)		67 (0.2)		280 (0.2)		173 (0.1)			83 (0.2)		256 (0.2)	
	Anesthesiology	156 (0.2)		27 (0.1)		183 (0.1)		38 (0)			8 (0)		46 (0)	
	Cardiology	5408 (5.7)		433 (1.3)		5841 (4.5)		6580 (5.4)			565 (1.2)		7145 (4.2)	
	Dermatology	32 (0)		16 (0)		48 (0)		317 (0.3)			50 (0.1)		367 (0.2)	
	Endocrinology	564 (0.6)		185 (0.5)		749 (0.6)		812 (0.7)			337 (0.7)		1149 (0.7)	
	Gastroenterology	8326 (8.7)		1418 (4.1)		9744 (7.5)		27,452 (22.5)			3163 (6.7)		30,615 (18.1)	
	General surgery	15,299 (16)		8275 (24)		23,574 (18.1)		10,878 (8.9)			12,273 (26.1)		23,151 (13.7)	
	Hematology/oncology	11,961 (12.5)		3828 (11.1)		15,798 (12.2)		22,419 (18.4)			4761 (10.1)		27,180 (16.1)	
	Infectious diseases	135 (0.1)		222 (0.6)		357 (0.3)		132 (0.1)			178 (0.4)		310 (0.2)	
	Internal medicine	931 (1)		380 (1.1)		1,311 (1)		284 (0.2)			225 (0.5)		509 (0.3)	
	Nephrology	1477 (1.5)		468 (1.4)		1945 (1.5)		1606 (1.3)			1138 (2.4)		2744 (1.6)	
	Neurology	3147 (3.3)		621 (1.8)		3768 (2.9)		2102 (1.7)			972 (2.1)		3074 (1.8)	
	Psychiatry	258 (0.3)		1037 (3)		1295 (1)		832 (0.7)			1478 (3.1)		2310 (1.4)	
	Neurosurgery	6101 (6.4)		5200 (15.1)		11,301 (8.7)		4391 (3.6)			2300 (4.9)		6691 (4)	
	Obstetrics/gynecology	15,137 (15.9)		1484 (4.3)		16,621 (12.8)		10,564 (8.6)			3516 (7.5)		14,080 (8.3)	
	Ophthalmology	891 (0.9)		32 (0.1)		923 (0.7)		5007 (4.1)			169 (0.4)		5176 (3.1)	
	Orthopedic surgery	8235 (8.6)		3158 (9.2)		11,393 (8.8)		3926 (3.2)			5382 (11.4)		9308 (5.5)	
	Otolaryngology	5712 (6)		492 (1.4)		6204 (4.8)		5470 (4.5)			777 (1.7)		6247 (3.7)	
	Pediatrics	153 (0.2)		28 (0.1)		181 (0.1)		1084 (0.9)			715 (1.5)		1799 (1.1)	
	Physiatry	208 (0.2)		962 (2.8)		1170 (0.9)		61 (0.1)			233 (0.5)		294 (0.2)	
	Plastic surgery	749 (0.8)		1056 (3.1)		1805 (1.4)		1802 (1.5)			1572 (3.3)		3374 (2)	
	Pulmonary disease	3098 (3.2)		540 (1.6)		3638 (2.8)		2772 (2.3)			1002 (2.1)		3774 (2.2)	
	Rheumatology	246 (0.3)		172 (0.5)		418 (0.3)		1041 (0.9)			458 (1)		1499 (0.9)	
	Thoracic surgery	3430 (3.6)		2576 (7.5)		6006 (4.6)		2584 (2.1)			3233 (6.9)		5817 (3.4)	
	Urology	3112 (3.3)		1610 (4.7)		4722 (3.6)		7721 (6.3)			1446 (3.1)		9167 (5.4)	
**Admission hour, n (%)**														
	9-12	15,280 (16)		6215 (18)		21,495 (16.5)		12,492 (10.2)			6352 (13.5)		18,844 (11.1)	
	12-15	57,431 (60.1)		19,892 (57.8)		77,323 (59.5)		53,044 (43.4)			22,512 (47.9)		75,556 (44.7)	
	15-18	19,401 (20.3)		7253 (21.1)		26,554 (20.5)		43,135 (35.3)			14,631 (31.1)		57,766 (34.1)	
	18-21	3387 (3.5)		1079 (3.1)		4466 (3.4)		13,466 (11)			3533 (7.5)		16,999 (10)	
**Day of week of hospital admission, n (%)**														
	Monday	20,048 (21)		6759 (19.6)		26,807 (20.6)		24,266 (19.9)			9399 (20)		33,665 (19.9)	
	Tuesday	16,790 (17.6)		6758 (19.6)		23,548 (18.1)		20,142 (16.5)			8966 (19.1)		29,108 (17.2)	
	Wednesday	16,914 (17.7)		6546 (19)		23,460 (18.1)		20,641 (16.9)			8833 (18.8)		29,474 (17.4)	
	Thursday	15,896 (16.6)		4733 (13.7)		20,629 (15.9)		20,027 (16.4)			5733 (12.2)		25,760 (15.2)	
	Friday	6987 (7.3)		2867 (8.3)		9854 (7.6)		8374 (6.9)			3109 (6.6)		11,483 (6.8)	
	Saturday	3116 (3.3)		1213 (3.5)		4329 (3.3)		3801 (3.1)			1905 (4.1)		5706 (3.4)	
	Sunday	15,748 (16.5)		5563 (16.2)		21,311 (16.4)		24,886 (20.4)			9083 (19.3)		33,969 (20.1)	
	Surgical operation, yes	45,776 (47.6)		23,850 (69.3)		69,626 (53.6)		30,128 (24.7)			22,164 (47.1)		52,292 (30.9)	
**Severity scores, mean (SD)**														
	CHADS_2_^d^VASc^e^	1.3 (1.1)		1.4 (1.2)		1.3 (1.1)		1.3 (1.1)			1.4 (1.2)		1.3 (1.1)	
	CHADS_2_	0.6 (1)		0.7 (1.1)		0.6 (1)		0.6 (1)			0.6 (1)		0.6 (1)	
	DCSI^f^	0.5 (1.2)		0.6 (1.4)		0.6 (1.3)		0.7 (1.3)			0.7 (1.4)		0.7 (1.3)	
	CCI^g^	2.1 (2.5)		2.2 (2.2)		2.11 (2.4)		2.6 (2.6)			2.5 (2.4)		2.6 (2.5)	

^a^SNUBH: Seoul National University Bundang Hospital.

^b^SNUH: Seoul National University Hospital.

^c^LoS: length of stay.

^d^CHADS_2_: clinical prediction tool used to test congestive heart failure, hypertension, age, diabetes, and stroke.

^e^VASc: The expanded version of CHADS_2_ that also tests for transient ischemic attack, vascular disease, age, and sex category.

^f^DSCI: Diabetes Comorbidity Severity Index.

^g^CCI: Romano’s adaptation of the Charlson Index.

### Performance in Internal Validation

**Figure 2:**
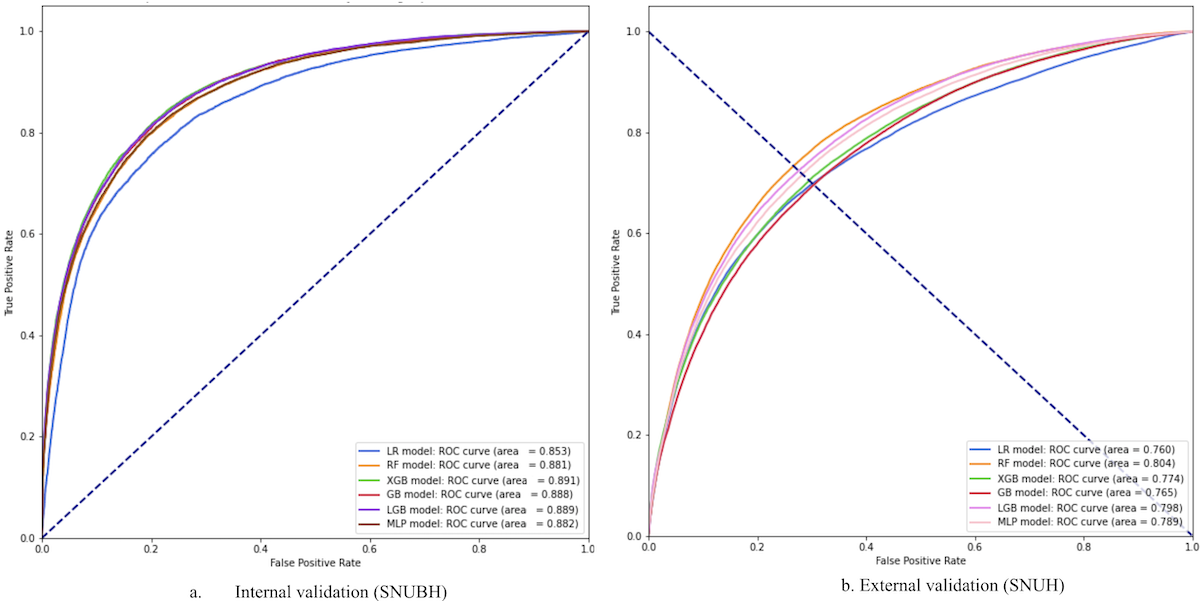
Receiver operating characteristics curves (AUROCs) for length of stay binary classification in the internal validation set. A. Internal validation was conducted at Seoul National University Bundang Hospital (SNUBH) B. External validation was conducted at Seoul National University Hospital (SNUH). CDM: Common Data Model; LoS: length of stay. LR: logistic regression; RF: random forest; XGB: Extreme Gradient Boosting; GB: Gradient Boosting; LGB: Light Gradient Boosting; MLP: Multilayer Perceptron. ROC: receiver operating characteristic.

In the internal validation of the prediction model, the XGB model achieved the top performance in planned admissions for binary classification, with an AUROC of 0.891 (95% CI 0.887-0.894) and an AUPRC of 0.819 (95% CI 0.813-0.826) (Table 2). The LGB model showed the highest performance in the multiclassification, with an AUROC of 0.901 (95% CI 0.898-0.904), 0.836 (95% CI 0.831-0.841), 0.750 (95% CI 0.742-0.758), 0.766 (95% CI 0.757-0.774), and 0.890 (95% CI 0.887-0.893) for LoS 3, 4, 5, 6, and ≥ 7 days, respectively, and a macro AUPRC of 0.556 (Table 3). Overall, the predictive performance for LoS consistently showed good discriminatory ability, achieving an AUROC >0.800 in both the binary and multiclassification scenarios (Figure 2A, Table 2).

**Table 2:** Model performance for binary outcomes in the internal and external validation sets. All numbers are presented with 95% CI.

Data set and models		AUROC^a^	AUPRC^b^	Sensitivity	Specificity	PPV^c^	NPV^d^
**Internal validation (n=38,847)**							
	LR^e^	0.853 (0.850-0.857)	0.744 (0.736-0.752)	0.771 (0.764-0.778)	0.786 (0.780-0.790)	0.642 (0.634-0.650)	0.873 (0.869-0.877)
	RF^f^	0.881 (0.878-0.885)	0.802 (0.795-0.808)	0.604 (0.596-0.613)	0.922 (0.918-0.925)	0.794 (0.786-0.801)	0.824 (0.819-0.828)
	XGB^g^	0.891 (0.887-0.894)	0.819 (0.813-0.826)	0.686 (0.679-0.695)	0.896 (0.893-0.900)	0.768 (0.760-0.775)	0.852 (0.848-0.856)
	GB^h^	0.888 (0.884-0.891)	0.811 (0.804-0.818)	0.681 (0.673-0.690)	0.894 (0.891-0.898)	0.763 (0.755-0.770)	0.849 (0.845-0.853)
	LGB^i^	0.889 (0.886-0.893)	0.816 (0.811-0.824)	0.661 (0.653-0.669)	0.906 (0.902-0.910)	0.778 (0.770-0.786)	0.843 (0.839-0.847)
	MLP^j^	0.882 (0.878-0.885)	0.804 (0.798-0.811)	0.635 (0.627-0.643)	0.910 (0.906-0.913)	0.778 (0.770-0.786)	0.833 (0.829-0.838)
**External validation (n = 169,165)**							
	LR	0.760 (0.757-0.763)	0.508 (0.503-0.514)	0.630 (0.622-0.632)	0.775 (0.773-0.777)	0.445 (0.441-0.449)	0.878 (0.876-0.880)
	RF	0.804 (0.802-0.807)	0.545 (0.540-0.551)	0.412 (0.408-0.417)	0.923 (0.921-0.924)	0.605 (0.601-0.611)	0.845 (0.843-0.846)
	XGB	0.774 (0.772-0.777)	0.523 (0.518-0.529)	0.473 (0.468-0.479)	0.879 (0.877-0.881)	0.530 (0.524-0.535)	0.853 (0.851-0.855)
	GB	0.765 (0.763-0.768)	0.499 (0.493-0.504)	0.481 (0.476-0.486)	0.863 (0.861-0.865)	0.503 (0.498-0.508)	0.852 (0.850-0.854)
	LGB	0.798 (0.796-0.801)	0.542 (0.537-0.548)	0.492 (0.487-0.497)	0.888 (0.887-0.890)	0.560 (0.555-0.565)	0.859 (0.857-0.860)
	MLP	0.789 (0.786-0.791)	0.532 (0.527-0.538)	0.449 (0.444-0.454)	0.890 (0.898-0.901)	0.563 (0.558-0.569)	0.850 (0.848-0.852)

^a^AUROC: area under the receiver operating characteristic curve.

^b^AUPRC: area under the precision-recall curve.

^c^PPV: positive predictive value.

^d^NPV: negative predictive value.

^e^LR: logistic regression.

^f^RF: random forest

^g^XGB: Extreme Gradient Boosting.

^h^GB: Gradient Boosting.

^i^LGB: Light Gradient Boosting.

^k^MLP: Multilayer Perceptron.

**Table 3:** Model performance for multiclass classification in the internal and external validation sets. All numbers are presented with a 95% CI.

Model and class		Internal validation set (n=38,847)					External validation set (n=169,165)					
		AUROC^a^	AUPRC^b^	Macroaveraged AUPRC		AUROC		AUPRC		Macroaveraged AUPRC		
**LR^c^**					0.486							0.342
	day 3 (class 0)	0.861 (0.857-0.866)	0.693 (0.683-0.702)			0.718 (0.715-0.720)		0.546 (0.542-0.550)				
	day 4 (class 1)	0.805 (0.799-0.811)	0.543 (0.531-0.555)			0.697 (0.694-0.700)		0.340 (0.335-0.345)				
	day 5 (class 2)	0.714 (0.705-0.722)	0.216 (0.206-0.229)			0.571 (0.567-0.576)		0.139 (0.136-0.143)				
	day 6 (class 3)	0.710 (0.700-0.719)	0.226 (0.213-0.240)			0.596 (0.591-0.601)		0.115 (0.112-0.118)				
	day 7 ≥ (class 4)	0.853 (0.849-0.857)	0.754 (0.746-0.762)			0.751 (0.748-0.754)		0.570 (0.566-0.575)				
**RF^d^**					0.534							0.379
	day 3 (class 0)	0.883 (0.880-0.887)	0.751 (0.742-0.759)			0.754 (0.751-0.756)		0.620 (0.616-0.625)				
	day 4 (class 1)	0.822 (0.817-0.828)	0.588 (0.576-0.600)			0.720 (0.717-0.723)		0.366 (0.361-0.371)				
	day 5 (class 2)	0.737 (0.729-0.745)	0.250 (0.237-0.264)			0.589 (0.585-0.594)		0.158 (0.154-0.162)				
	day 6 (class 3)	0.751 (0.742-0.760)	0.296 (0.280-0.312)			0.627 (0.622-0.632)		0.138 (0.134-0.142)				
	day 7 ≥ (class 4)	0.872 (0.869-0.875)	0.784 (0.778-0.791)			0.797 (0.794-0.799)		0.613 (0.608-0.617)				
**XGB^e^**					0.546							0.363
	day 3 (class 0)	0.899 (0.896-0.903)	0.770 (0.761-0.778)			0.753 (0.751-0.755)		0.609 (0.605-0.613)				
	day 4 (class 1)	0.829 (0.825-0.835)	0.595 (0.583-0.606)			0.703 (0.700-0.706)		0.343 (0.338-0.348)				
	day 5 (class 2)	0.735 (0.727-0.744)	0.251 (0.239-0.265)			0.570 (0.565-0.574)		0.135 (0.131-0.138)				
	day 6 (class 3)	0.751 (0.742-0.760)	0.298 (0.282-0.315)			0.600 (0.594-0.604)		0.123 (0.119-0.126)				
	day 7 ≥ (class 4)	0.887 (0.884-0.891)	0.814 (0.808-0.821)			0.785 (0.782-0.787)		0.607 (0.602-0.611)				
**GB^f^**					0.538						0.326	
	day 3 (class 0)	0.900 (0.896-0.903)	0.766 (0.757-0.774)			0.718 (0.716-0.721)		0.562 (0.558-0.567)				
	day 4 (class 1)	0.829 (0.824-0.835)	0.593 (0.580-0.605)			0.621 (0.618-0.624)		0.253 (0.249-0.257)				
	day 5 (class 2)	0.735 (0.727-0.743)	0.236 (0.225-0.248)			0.600 (0.595-0.604)		0.146 (0.143-0.150)				
	day 6 (class 3)	0.754 (0.745-0.762)	0.285(0.270-0.301)			0.564 (0.559-0.569)		0.103 (0.101-0.106)				
	day 7 ≥ (class 4)	0.888 (0.885-0.891)	0.812 (0.806-0.819)			0.754 (0.751-0.756)		0.565 (0.560-0.570)				
**LGB^g^**					0.556							0.383
	day 3 (class 0)	0.901 (0.898-0.904)	0.770 (0.762-0.779)			0.766 (0.764-0.768)		0.626 (0.622-0.630)				
	day 4 (class 1)	0.836 (0.831-0.841)	0.606 (0.594-0.618)			0.724(0.721-0.727)		0.361 (0.375-0.386)				
	day 5 (class 2)	0.750 (0.742-0.758)	0.270 (0.256-0.284)			0.595 (0.590-0.599)		0.146 (0.143-0.149)				
	day 6 (class 3)	0.766 (0.757-0.774)	0.318 (0.302-0.335)			0.616 (0.611-0.621)		0.132 (0.128-0.135)				
	day 7 ≥ (class 4)	0.890 (0.887-0.893)	0.818 (0.812-0.824)			0.808 (0.806-0.810)		0.632 (0.628-0.637)				
**MLP^h^**					0.523							0.346
	day 3 (class 0)	0.887 (0.883-0.890)	0.736 (0.727-0.745)			0.735 (0.732-0.737)		0.582 (0.578-0.586)				
	day 4 (class 1)	0.821 (0.816-0.827)	0.574 (0.561-0.586)			0.685 (0.682-0.689)		0.333(0.328-0.338)				
	day 5 (class 2)	0.730 (0.722-0.739)	0.239 (0.228-0.253)			0.590 (0.586-0.594)		0.137 (0.134-0.140)				
	day 6 (class 3)	0.736 (0.727-0.745)	0.276 (0.260-0.291)			0.603 (0.598-0.608)		0.120 (0.117-0.123)				
	day 7 ≥ (class 4)	0.874 (0.871-0.878)	0.791 (0.784-0.798)			0.747(0.745-0.750)		0.559 (0.554-0.564)				

^a^AUROC: area under the receiver operating characteristic curve.

^b^AUPRC: area under the precision-recall curve.

^c^LR: logistic regression.

^d^RF: random forest

^e^XGB: Extreme Gradient Boosting.

^f^GB: Gradient Boosting.

^g^LGB: Light Gradient Boosting.

^h^MLP: Multilayer Perceptron.

### SHAP Calibration Plots and Brier Scores in Internal Validation

**Figure 3:**
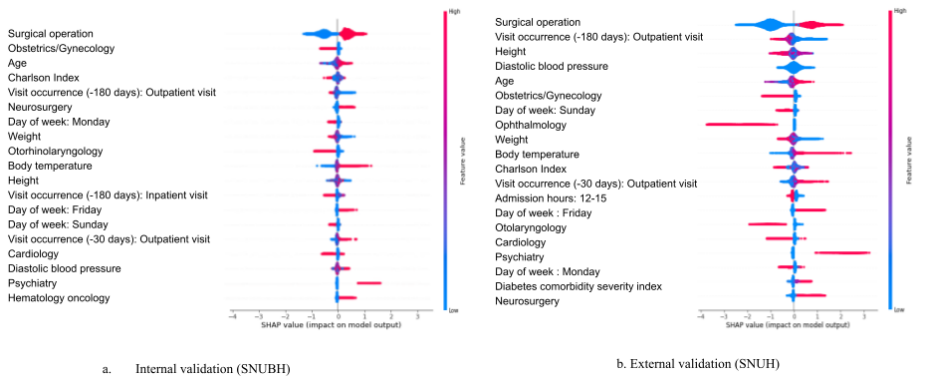
Shapley Additive Explanations (SHAP) feature importance analysis for the model predicting length of stay of 7 days or longer. A. Internal validation was conducted at Seoul National University Bundang Hospital (SNUBH) B. External validation was conducted at Seoul National University Hospital (SNUH).

**Figure 4:**
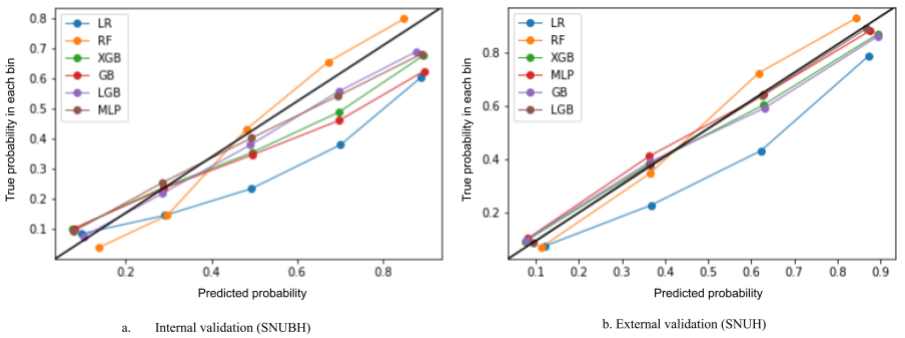
Calibration of length of stay prediction models for 7 days or longer in internal and external validation sets. A. Internal validation was conducted at Seoul National University Bundang Hospital (SNUBH) B. External validation was conducted at Seoul National University Hospital (SNUH). LR: logistic regression; RF: random forest; XGB: Extreme Gradient Boosting; GB: Gradient Boosting; LGB: Light Gradient Boosting; MLP: Multilayer Perceptron.

The most important features contributing to the predictive performance of the models were the operation performed, the admitted patient’s department, age, and severity scores such as the Charlson Index-Romano adaptation and the frequency of outpatient visits in the past 6 months (Figure 3A). In the multiclassification models, the SHAP analysis revealed distinct predictors, including surgical operations, blood pressure, and day of the week of hospital admission, which significantly influenced different hospital stay durations (Figure S2 in Multimedia Appendix 1). Specifically, surgical operations were significant predictors for LoS of 3 days and 7 days or longer, while admissions in obstetrics/gynecology and neurosurgery were relevant for LoS of 4 and 5 days. Additionally, hospital admissions on Wednesdays and Fridays were associated with a 6-day LoS (Figure S2 in Multimedia Appendix 1). The calibration plot and Brier scores indicated strong calibration performance, achieving approximately 0.14 scores in both the binary and multiclassification models (Figure 4A, Table S3 in Multimedia Appendix 1).

### Performance in the External Validation

In the external validation, the RF model performed the best in predicting an LoS of 7 days or longer with an AUROC of 0.804 (95% CI 0.802-0.807) and an AUPRC of 0.545 (95% CI 0.540-0.551) (Table 3). The AUROC decreased by 8% compared to the AUROC obtained from SNUBH (Figure 2B). The LGB model showed superior overall performance for multiclassification, with an average AUROC of 0.711 and a macro AUPRC of 0.383 across different LoS durations (Table 3). The AUROC showed a 0.93 reduction compared to the internal validation multiclassification model.

### SHAP, Calibration Plots, and Brier Scores in the External Validation

The SHAP analysis identified significant predictors for hospital stays of 7 days or longer, including surgical procedures, elevated body temperatures, frequency of outpatient visits in the past month, hospital admissions on Fridays, and older age (Figure 3B). In the multiclassification analysis, akin to the prediction model’s internal validation, cases with surgery performed showed an increased LoS in the hospital. Admission to the cardiology department generally led to discharge within 3 days. Specifically, admissions to the obstetrics/gynecology and hematology/oncology departments were associated with an average LoS of 4 and 5 days, respectively. Hospital admissions on Sundays and a higher number of outpatient visits in the past month resulted in hospital stays lasting more than 6 days (Figure S3 in Multimedia Appendix 1). The binary and multiclassification models accurately calibrated predicted probabilities and actual outcomes on the external validation data set (Figure 4B, Table S4 in Multimedia Appendix 1).

## Discussion

### Principal Findings

In this paper, we proposed two ML models using the standardized vocabularies of the OMOP CDM: (1) a binary classification model predicting LoS of 7 days or more and (2) a multiclassification model categorizing LoS as 3, 4, 5, 6, and 7 days or longer. This study demonstrates that combining clinical and nonclinical characteristics from a standardized data source from different institutions yields moderate predictive accuracy and efficiently leverages the benefits of multicenter studies. To our knowledge, our model is the first to offer both binary and multiclass classification predictions for LoS using the OMOP CDM.

The ML algorithms using GB performed the highest binary classification performance, while the LGB model performed the best in the multiclass classification. The ML predictions identified surgical operation, diastolic blood pressure, and the day of week of hospital admissions as critical factors for predicting both an LoS of 7 days or more and for multiclass classification of LoS. The external validation revealed a model performance 8% lower than the internally calculated AUROC value. This decline in performance could be attributed to variations in patient populations and medical specialties across hospitals and disparities in converting EHR data into the standardized OMOP CDM format [29-31].

In this study, we conducted an extensive analysis across 46 departments, developing a model that predicted LoS from 3 to 7 days or longer. A multidepartment approach unraveled the complex dynamics of patient stays and illuminated the varied factors influencing each day’s length of stay. Insights from this broad patient spectrum could equip health care professionals with essential tools for improving patient care strategies in various health care settings. These models may aid in optimizing health care resources and contribute to identifying patients most likely to be discharged on a particular day using classification algorithms.

Additionally, our LOS prediction model offers potential economic benefits by enabling better planning and management of bed occupancy, reducing costs associated with the inefficient use of hospital beds. By anticipating extended stays, hospitals can arrange necessary interventions in advance, potentially preventing prolonged hospitalizations and lowering treatment costs. Streamlining discharge processes based on accurate predictions reduces delays and frees up beds more quickly, leading to cost savings in terms of staffing and resource management. Although further studies are needed to empirically validate these benefits, our findings suggest that implementing such predictive models could support clinical and financial improvements in hospital management.

Earlier studies often used data sources such as administrative data, the MIMIC, and EHRs for model development, resulting in limited applicability across different institutions due to heterogeneous data standards (Table S1 in Multimedia Appendix 1). The variations in these data sources hindered scalability and the capacity to efficiently manage a wide range of routinely collected observational health data. Previously utilized data constrained the transferability of the model outputs to different patient populations across multiple medical specialties and institutions [5,17,32]. We designed prediction models that implemented the OMOP CDM standardized vocabularies and demonstrated their application across independent institutions, ensuring interoperability and applicability. Although one study predicted LoS from multicenter data using federated learning, it relied solely on administrative data and trained with 3 regression models, suggesting multicollinearity issues [33]. Unlike the aforementioned model, ours included hospital stay characteristics and patients’ medications incorporating more records than the federated learning research. As a result, our model, including hospital admission information and comprehensive patient history, provides more reliable insights, covering a broader range of records.

Systematic reviews assessing efficacious health system interventions to avoid prolonged hospital LoS in high-risk populations indicate that reducing LoS requires a multifaceted approach, including clinical care and the logistics of care coordination [4]. However, most previous studies primarily used demographics, lab results, and vital signs as input features [1]. Some authors integrated data from different modalities, combining free-text notes and demographic information with time-varying features, while only a few employed medications as predictive variables [1]. In this study, we incorporated clinical and nonclinical features, including medical specialties, severity scores, day and time of admission, and hospital admissions during the weekend. Time-dependent covariates classified as occurring in the past 1, 6, and 12 months include frequency of different types of visits, active ingredients in a drug group, and medical conditions to capture health care utilization patterns. This comprehensive data integration provides a longitudinal view of the patient, enabling more personalized care planning and effective discharge strategies tailored to each patient’s recovery.

When comparing prediction models for a 7-day LoS using the OMOP CDM within the 2 tertiary hospitals, we found that surgical operation, admitting unit, patient age, severity scores, and admission day significantly influenced hospital stay duration. For example, our internal validation revealed that older patients admitted to the neurosurgery department and undergoing surgery often have longer LoS, aligning with the notion that older adult patients requiring neurosurgery tend to have poorer prognosis and generally require extended hospitalization [34]. In contrast, admissions to obstetrics/gynecology appeared to have a shorter LoS, likely because they are mostly elective surgeries, such as cesarean deliveries [35,36]. A lower frequency of outpatient visits in the past 6 months was associated with a longer LoS, indicating that patients who visit the hospital less frequently may not be managing their health effectively. Friday admissions, typically through the emergency room, were more likely to involve severe conditions, while the unavailability of elective surgeries during the weekend could result in a longer LoS [37]. Weekend admissions, particularly on Sundays and Mondays, were associated with shorter LoS, likely due to the scheduling of preplanned treatments and elective surgeries on weekdays [38]. Additionally, certain medical specialties were linked to extended LoS. Psychiatric admission often resulted in longer stays, attributed to the need for comprehensive assessments and stabilization in mental health care [39]. Similarly, admissions to hematology/oncology departments frequently reported prolonged stays for monitoring and managing side effects and complications arising from hematologic cancer treatments [40]. Higher body temperatures indicated more severe conditions, potentially leading to longer durations of illness [41].

### Limitations

This study has several limitations. The models were only externally validated in a single hospital, which may limit their applicability to other health care settings with different patient populations and characteristics. However, health care systems with OMOP CDM can readily implement the methods proposed in this study using standardized vocabularies and analytic codes [17,23,42]. This work aids in developing a model that predicts the remaining LoS based on hospitalization progression during the admission period within a heterogeneous population through the Observational Health Data Sciences and Informatics (OHDSI) network study. Additionally, smaller patient numbers in the 5- and 6-day LoS categories in both the internal and external validation sets (Table S5 in Multimedia Appendix 1) may have affected the model performance for these subgroups. The models may not fully capture real-world performance due to the limited consideration of local context and resources such as team dynamics, hospital processes, staffing resources, social drivers of health, and administrative support—limitations stemming from nonstandard fields in the OMOP CDM. Using the standardized information we could capture from the CDM, we developed a reproducible model based on standardized variables that are less influenced by individual hospitals, such as clinical factors and hospital stay characteristics.

### Conclusion

In this study, we utilized the OMOP CDM to predict the LoS in planned admissions, considering both binary (LoS ≥ 7 days) and multiclassification scenarios (LoS of 3, 4, 5, 6, and 7 days or more). The performance of the prediction models was consistent and confirmed through external validation. The proposed models showed ease of application in various clinical settings and reproducibility across institutions within the OHDSI community. The findings of this study may assist hospitals in effectively managing hospital resources, such as staffing, equipment, and supplies at different institutions.

## Appendix

Multimedia Appendix 1 Supplementary material for the study.

## Abbreviations

AUROC: area under the receiver operating characteristic curve
AUPRC: area under the precision-recall curve
ED: emergency department
EHR: electronic health record
ETL: extract, transform, and load
GB: Gradient Boosting
LGB: Light Gradient Boosting
LR: logistic regression
ML: machine learning
MIMIC: Medical Information Mart for Intensive Care
OHDSI: Observational Health Data Sciences and Informatics
OMOP CDM: Observational Medical Outcomes Partnership Common Data Model
RF: random forest
SHAP: Shapley Additive Explanations
SNUH: Seoul National University Hospital
SNUBH: Seoul National University Bundang Hospital
XGB: Extreme Gradient Boosting

## Data Availability

Because this study utilized a distributed research network, the data are kept within each institution and are not publicly available. However, the source code for cohort and covariates generation is available in the GitHub repository [43].
