# Supplementary material for: Hospital Length of Stay Prediction for Planned Admissions Using Observational Medical Outcomes Partnership Common Data Model: Retrospective Study
Source: J Med Internet Res. 2024 Nov 22;26:e59260. doi: 10.2196/59260 (PMC11624451; doi:10.2196/59260)
Supplement: Multimedia Appendix 1 [file jmir_v26i1e59260_app1.docx]

**Appendix 1**

Hospital Length of Stay Prediction for Planned Admissions Using OMOP CDM

**Table of Contents Page (s)**

**Supplementary Table S1.** Literature review summary Page 2-9

**Supplementary Table S2.** List of Statistical and Analytical Packages Used in the Study 10

**Supplementary Table S3.** Calculation of brier scores for multi-class classification in the internal validation set 11-12

**Supplementary Table S4.** Calculation of brier scores for multi-class classification in the external validation set 13-14

**Supplementary Table S5.** Patient distribution across hospital length of stay categories in internal 15

and external validation sets

**Supplementary Figure S1.** Distribution of Length of Stay across admission departments in the internal validation set 16

**Supplementary Figure S2.** SHAP analysis for multi-class prediction using the internal validation set 17

**Supplementary Figure S3.** SHAP analysis for multi-class prediction using the external validation set 18

| **Supplementary table S1. Literature review summary** | | | | | | | | | |
| --- | --- | --- | --- | --- | --- | --- | --- | --- | --- |
| **Author** | **Study Setting** | **Target population** | **Hospital admissions** | **Data source** | **No. patients** | **Outcome** | **Model** | **Internal validation** | **External validation** |
| [Cai et al (2016)](https://pubmed.ncbi.nlm.nih.gov/26374704/) | Hospital-based | ER visits | Unplanned patients | EHR | 32,634 | LoS : 0-6 days LoS >=7 days" | Bayesian Network | AUROC 0.76 | NA |
| [Launay et al (2015)](https://www.ejinme.com/article/S0953-6205(15)00207-1/fulltext) | Hospital-based | ER visits | Unplanned patients | EHR | 993 | LoS>=13 days | ANN  MLP | AUROC 0.905 | NA |
| [Alghatani et al (2021)](https://medinform.jmir.org/2021/5/e21347/) | Multi-center | ICU patient | Planned patients | MIMIC | 44,626 | ICU Length of stay (LoS>2.636 days) | LR, RF, KNN, SVM, XGB, MLR, SVR | AUROC 0.70 | NA |
| [Usher et al (2021)](https://academic.oup.com/jamiaopen/article/4/3/ooab055/6317834) | Multi-center | Patients with COVID-19 | Planned patients | EHR | 2,086 | LoS (>5, >10, >15 days) | GLM | AUROC 0.890 | NA |
| [Barsaella et al (2022)](https://www.mdpi.com/1648-9144/58/11/1568) | Nationwide | Diabetes and hypertension inpatients | Planned patients | Claims | 58,618 | LoS | SVM, LR, GBM, XGBoost, RF | RSME 0.386 | NA |
| [Rajkomar et al (2018)](https://www.nature.com/articles/s41746-018-0029-1) | Multi-center | General inpatients | Planned patients | EHR (free text) | 114,003 | LoS ≤ 7 days | LSTM, TANN | AUROC 0.86 | NA |
| [Abujaber et al (2022)](https://www.sciencedirect.com/science/article/pii/S2666521222000059) | Multi-center | Patients with traumatic brain injury | Planned patients | Trauma registry | 1,417 | LoS >=23 days | ANN | AUROC 0.915 | NA |
| [Wu et al (2021)](https://pdfs.semanticscholar.org/068e/1317a80cb99c00d58cbab892818420eedde7.pdf) | Multi-center | ICU patients | Planned patients | eICU | 117,306 | ICU LoS >=3 days | RF, SVM, GBDT, |  |  |
| [Ma et al (2020)](https://pubmed.ncbi.nlm.nih.gov/31765937/) | Hospital-based | ICU patients | Planned patients | PhysioNet | 4,000 | ICU LoS >=10 days | JITL and one-class ELM | AUROC 0.85 | NA |
| [Zhang et al (2020)](https://www.ncbi.nlm.nih.gov/pmc/articles/PMC7596962/) | Multi-center | ICU patients | Planned patients | MIMIC-III | 39,429 | LoS >7 days | LR, RF, Fusion-LSTM, Fusion-CNN | AUROC 0.87 |  |
| [Zebin et al (2019)](https://ieeexplore.ieee.org/document/8791477) | Multi-center | ICU patients | Planned patients | MIMIC-III | 53,404 | LoS : 0-7 days LoS >7 days | Autoencoder+DNN model | ACC 77.7% | NA |
| [Wang et al (2020)](https://arxiv.org/pdf/1907.08322.pdf) | Multi-center | ICU patients | Planned patients | MIMIC-III | 34,472 | LoS > 3 days ICU LoS > 7 days | LR, RF, GRU-D | AUROC 0.736 (LoS > 3 days) AUROC 0.764 (LoS > 7 days) | NA |
| [Alsinglawi et al (2020)](https://research.uaeu.ac.ae/en/publications/benchmarking-predictive-models-in-electronic-health-records-sepsi) | NA | Patients with sepsis | Planned patients | EHR, MIMIC-III | 61,532 admissions | LoS > 7 days | RF | AUROC 0.93 | NA |
| [Alahmar et al (2018)](https://ieeexplore.ieee.org/stamp/stamp.jsp?tp=&arnumber=8500068) | Multi-center | Patients with diabetes | Planned patients | HER | 43,092 | LoS ≤ 3 days | NB, GLM, Deep learning, RF, GB, stacked ensemble | AUROC 0.81 | NA |
| [Gong et al (2017)](https://dl.acm.org/doi/10.1145/3097983.3098064) | Multi-center | ICU patients | Planned patients | MIMIC-III | 28,493 | ICU LoS >= 8 days | LR + NLP | AUROC 0.77 | NA |
| [Huang et al (2019)](https://www.sciencedirect.com/science/article/pii/S1532046419302102) | Multi-center | ICU patients | Planned patients | eICU | 28,000 | ICU LoS >= 8 days | community-based federated machine learning-5 | AUROC 0.651 | NA |
| [Gentimis et al (2017)](https://www.researchgate.net/publication/324177552_Predicting_Hospital_Length_of_Stay_Using_Neural_Networks_on_MIMIC_III_Data) | Multi-center | ICU patients | Planned patients | MIMIC-III | 50,000 | LoS > 5 days | NN, RF | ACC 79% | NA |
| [Jalai et al (2020)](https://www.nature.com/articles/s41598-020-62971-3) | Multi-center | Patients with cardiac transplantation | Planned patients | Pediatric Heart Network repository | 664 | LoS > 41 days | - LR - DT - RF - GB - DNN | AUROC 0.94 | NA |
| [Weissman et al (2018)](https://www.ncbi.nlm.nih.gov/pmc/articles/PMC6005735/pdf/nihms951452.pdf) | Multi-center | ICU patients | Planned patients | MIMIC-III | 25,947 | ICU LoS > 7 days ICU LoS > 21 days | - LR - GB - RF - Elastic net regression | AUROC 0.890 (a) AUROC 0.84 (b) | NA |
| [Harerimana et al (2021)](https://www.sciencedirect.com/science/article/pii/S1532046421001076) | Multi-center | ICU patients | Planned patients | MIMIC-III | 47,796 | LoS ≤ 10 days 10 <LoS ≤ 30 LoS > 30 days | hierarchical attention network | AUROC 0.821 | NA |
| [Sanchez-Arias et al (2020)](https://www.semanticscholar.org/paper/A-Methodology-for-Estimating-Hospital-Intensive-of-Batista-Sanchez-Arias/be923a794f8f88a67d7b7b785d9c8d65793bc4ee) | Multi-center | ICU patients | Planned patients | MIMIC-III | 61,293 | ICU LoS ≤ 3 days 3 days < ICU LoS < 10 days ICU LoS ≥ 10 days | - RF - RF ranger - SVM - SVM_rbf - boost_tree - SVM + GBT + DT | ACC: 0.927 | NA |
| [Ramkumar et al (2019)](https://pubmed.ncbi.nlm.nih.gov/31122849/) | Nationwide | Patients with primary total hip arthroplasty cases for osteoarthritis | Planned patients | Adminstrative data | 78,335 | 1 day ≤LoS ≤ 2 days 3 days ≤ LoS ≤ 5 days LoS ≥ 6 days | - NB - ANN | AUROC 0.820 | AUROC 0.803 |
| [Navarro et al (2018)](https://pubmed.ncbi.nlm.nih.gov/30243882/) | Nationwide | Patients with primary total knee arthroplasty | Planned patients | Adminstrative data | 141,446 | 1 day ≤ LoS ≤ 3 days 4 ≤ LoS ≤ 5 days LoS > 5 days | NB | AUROC 0.782 | NA |
| [Alturki et al (2019)](https://www.researchgate.net/publication/339980149_Predictors_of_Readmissions_and_Length_of_Stay_for_Diabetes_Related_Patients) | Multi-center | Patients with diabetes | Unplanned patients | EHR | 101,766 | 1 day ≤ LoS ≤ 4 days 5 days ≤ LoS ≤ 8 days 9 days ≤ LoS ≤ 14 days | - LR - RF, - SVM, - XGB, - KNN, | ACC 0.88 | NA |
| [Karnuta et al (2019)](https://journals.lww.com/jorthotrauma/Abstract/2019/07000/Bundled_Care_for_Hip_Fractures__A_Machine_Learning.2.aspx) | Nationwide | Patients undergoing hip fracture surgery | Planned patients | Hospital Inpatient Discharges (SPARCS De-Identified) | 98,562 | 1 day ≤LoS ≤ 3 days 4 days ≤ LoS ≤ 6 days LoS ≥ 10 days | - NB with adpative boosting | AUROC 0.88 | NA |
| [Xu et al (2018)](https://dl.acm.org/doi/10.1145/3219819.3220051) | Multi-center | ICU patienst | Planned patients | MIMIC-III | 22,317 waveform records | ICU LoS of 1-7 days 8 days ≤ ICU LoS ≤ 14 days ICU LoS > 14 days | - Recurrent Attentive and Intensive Model - CNN-RNN - CNN-AttRNN | ACC 86.32% | NA |
| [Barnes et al (2016)](https://academic.oup.com/jamia/article/23/e1/e2/2379761) | Hospital-based | General inpatients | Planned patients | EHR | 8,852 | 2 PM At the end of day | - LR - Regression RF | Sensitivity 0.715 (end of day) Specificity 0.929 (end of day) | NA |
| [Haya et al (2017)](https://www.proquest.com/openview/6a5a990e1ec4f2728dfe913a5e47bf3a/1?pq-origsite=gscholar&cbl=18750) | Community-based | General inpatients | Planned patients | Hospital Inpatient Discharges (SPARCS De-Identified) | NA | LoS | - DT - KNN - NB - LR - SVM - MLP - RF - GB - DBN | ACC 88.5% | NA |
| [Jaotombo et al (2023)](https://www.ncbi.nlm.nih.gov/pmc/articles/PMC9707380/pdf/ZJMA_11_2149318.pdf) | Nationwide | ICU patienst | Planned + unplanned patients | French Medico-Administrative database | 73,182 | LoS >14 days | - GB - LR - CART - RF - GB - NN | AUROC 0.810 | NA |
| [Steele et al (2019)](https://ieeexplore.ieee.org/document/8666598) | Community-based | Elective admissions | Planned patients | Florida State Inpatient Database (SID) | 120,882 | LoS <8 days | - KNN - KStar - C4.5 DT - SVM - NB | AUROC 0.904 | NA |
| [Caetano et al (2015)](https://link.springer.com/chapter/10.1007/978-3-319-22348-3_9) | Hospital-based | General inpatients | Planned patients | EHR | 26,462 | LoS | - MLR - DT - ANN - SVM - RF | R2 0.813 RMSE 0.469 | NA |
| [Livieris et al (2018)](https://www.mdpi.com/1999-4893/11/12/199) | Hospital-based | ICU patienst | Planned patients | EHR | 2,702 | LoS : 1-5, 5+ days | - NB - MLP - KNN - RF - SMO - two-level classifier | ACC 78.5% | NA |
| [Liveris et al (2018)](https://www.researchgate.net/publication/326462980_Predicting_length_of_stay_in_hospitalized_patients_using_SSL_algorithms) | Hospital-based | ICU patienst | Planned patients | EHR | 4,403 | LoS : 1-2, 3-6, 6+ days | - NB - MLP - SMO - 3NN - C4.5 DT - PART | ACC 64.77% | NA |
| [Baek et al (2018)](https://www.ncbi.nlm.nih.gov/pmc/articles/PMC5898738/) | Hospital-based | General inpatients | Planned patients | EHR | 45,546 | LoS : >30 days | Multiple regression analysis | ACC 0.973 | NA |
| [Cui et al (2018)_](https://www.tandfonline.com/doi/abs/10.1080/24725579.2018.1512537) | Nationwide | General inpatients | Planned patients | EHR | 750,000 | LoS | - Multi-task lasso - DT - RF - NN | R2 0.554 | NA |
| [Vincent et al (2010)](https://journals.lww.com/lww-medicalcare/Fulltext/2010/08000/Length_of_Stay_Predictions__Improvements_Through.11.aspx) | Multi-center | General inpatients | Planned patients | EHR | 155,474 | LoS | - LR - generalized linear models | R2 0.146 | NA |
| [Bacchi et al (2021)](https://pubmed.ncbi.nlm.nih.gov/33728577/) | Hospital-based | General inpatients | Planned patients | EHR | 26,217 | LoS < 3 days LoS <7 days | - ANN - LR | AUROC 0.80 (LoS < 3 days) AUROC 0.48 (LoS < 7 days) | NA |
| [Levin et al (2020)](https://innovations.bmj.com/content/7/2/414) | Hospital-based | General inpatients | Planned patients | EHR | 120,780 (predictions) 12,470 (prospective study) | LoS (same day vs. next day) | Supervised ML | AUROC 0.7 - 0.8 | NA |
| [Kalgotra et al (2021)](https://www.tandfonline.com/doi/abs/10.1080/07421222.2021.1990618) | Multi-center | General inpatients | Planned patients | EMR | 10 million | LoS | - LSTM | R2 0.652 | NA |
| [Grampurohit et al (2020)](https://ieeexplore.ieee.org/stamp/stamp.jsp?tp=&arnumber=9298294) | Multi-center | General inpatients | Planned patients | MIMIC-II | 4,927 | LoS | - Linear, Ridge - Lasso - ElasticNet | MAE 0.951 | NA |
| [Zeng et al (2022)](https://www.hindawi.com/journals/cin/2022/9517029/) | Hospital-based | General inpatients | Planned patients | Hospital Inpatient Discharges (SPARCS De-Identified) | 2,343,569 instances | LoS | - LR - ridge regression - RF regression - Light GBM - XGBR | R2 0.960 MSE 2.231 | NA |
| [Walraven et al (2017)](https://shmpublications.onlinelibrary.wiley.com/doi/full/10.12788/jhm.2802) | Hospital-based | General inpatients | Planned patients | EHR | 192,859 | LoS | - proportional hazard regression model | R2 0.892 | NA |
| [McCoy et al (2018)](https://pubmed.ncbi.nlm.nih.gov/30646340/) | Two centers | General inpatients | Planned patients | EHR | Hospital A: 54, 411 Hospital B: 47,456 | LoS | - a seasonal autoregressive-integrated moving average (SARIMA) model - Last week carried forward - Last year carried forward - Mean of last week and year - Prophet | R2 0.843 | NA |
| [Bertsimas et al (2019)](https://www.medrxiv.org/content/medrxiv/early/2020/05/18/2020.05.12.20098848.full.pdf) | Hospital-based | General inpatients | Planned patients | EHR | 41,726 | LoS: 1, 2, 7, 14 days | - LR - DT - RF - GBT | AUROC 0.843 (remaining LoS <1 day) | Prospective study |

LR = Logistic Regression. SVM = Support Vector Machine. RF = Random Forest. XGB: Extreme Gradient Boosting. MLP: Multi-layer Perceptron. LGB: Light Gradient Boosting. AUROC: area under the receiver operating characteristic curve. AUPRC: area under the precision-recall curve. ACC: accuracy. ANN: Artificial Neural Network. MLR: multivariate linear regression. SVR: support vector regression. GLM: generalized linear model. LSTM : long short-term memory. TANN: Trained artificial neural network. GBDT: gradient boosting decision tree. JITL : just-in-time learning. one-class ELM: one-class extreme.

CNN: convolutional neural network. DNN: deep neural network. GRU-D: gated recurrent unit with delay. NB: Naive Bayes. GLM: Generalized linear model. DT: Decision Tree. CART: lassification and regression trees. KNN: k-nearest neighbors. SMO: Sequential minimum optimization

**Supplementary Table S2.** List of Statistical and Analytical Packages Used in the Study

| **Package** | |
| --- | --- |
| readxl | Pandas |
| Magrittr | Csv |
| Dplyr | SelectFromModel |
| moonBook | LogisticRegression |
| Table1 | Lasso |
| PatientLevelPrediction | KFold |
| Andromeda | Train_test_split |
| Devtools | MinMaxScaler |
| DBI | GroupShuffleSPlit |
| RPostgreSQL | Roc_auc_score |
| RJDBC | RandomizedSearchCV |
| rJava | StratifiedKFold |
| DatabaseConnector | F1_score |
| Cyclops | Auc |
| FeatureExtraction | Classification_report |
| SqlRender | Precision_score |
| Ggplot2 | Recall_score |
| numpy | Accuracy_score |
| Average_precision_score | Confusion_matrix |
| Precision_recall_curve | RandomForestClassifier |
| Cycle | XGBClassifier |
| Matplotlib.pyplot | GradientBoostingClassifier |
| Calibration_curve | LGBMClassifier |
| Seaborn | shap |
| Pickle | MLPClassifier |
| pandas | Brier_score_loss |

**Supplementary Table S3. Calculation of brier scores for multi-class classification in the internal validation set**

| **Class** | **Model** | **Brier Score** | |
| --- | --- | --- | --- |
| 3 days | LR | 0.13 |  |
|  | RF | 0.12 |  |
|  | XGB | 0.11 |  |
|  | LGB | 0.11 |  |
|  | GB | 0.11 |  |
|  | MLP | 0.12 |  |
| 4 days | LR | 0.12 |  |
|  | RF | 0.12 |  |
|  | XGB | 0.12 |  |
|  | LGB | 0.12 |  |
|  | GB | 0.12 |  |
|  | MLP | 0.12 |  |
| 5 days | LR | 0.09 |  |
|  | RF | 0.08 |  |
|  | XGB | 0.08 |  |
|  | LGB | 0.08 |  |
|  | GB | 0.09 |  |
|  | MLP | 0.08 |  |
| 6 days | LR | 0.08 |  |
|  | RF | 0.07 |  |
|  | XGB | 0.07 |  |
|  | LGB | 0.07 |  |
|  | GB | 0.07 |  |
|  | MLP | 0.07 |  |
| ≥ 7days | LR | 0.14 |  |
|  | RF | 0.14 |  |
|  | XGB | 0.12 |  |
|  | LGB | 0.12 |  |
|  | GB | 0.12 |  |
|  | MLP | 0.13 |  |

LR = Logistic Regression. RF = Random Forest. XGB: Extreme Gradient Boosting. Gradient Boosting. MLP: Multi-layer Perceptron. LGB: Light Gradient Boosting.

**Supplementary Table S4. Calculation of brier scores for multi-class classification in the external validation set**

| **Class** | **Model** | **Brier Score** |
| --- | --- | --- |
| 3 days | LR | 0.21 |
|  | RF | 0.19 |
|  | XGB | 0.19 |
|  | LGB | 0.19 |
|  | GB | 0.22 |
|  | MLP | 0.21 |
| 4 days | LR | 0.14 |
|  | RF | 0.14 |
|  | XGB | 0.14 |
|  | LGB | 0.13 |
|  | GB | 0.17 |
|  | MLP | 0.15 |
| 5 days | LR | 0.09 |
|  | RF | 0.09 |
|  | XGB | 0.1 |
|  | LGB | 0.35 |
|  | GB | 0.09 |
|  | MLP | 0.1 |
| 6 days | LR | 0.08 |
|  | RF | 0.07 |
|  | XGB | 0.08 |
|  | LGB | 0.08 |
|  | GB | 0.07 |
|  | MLP | 0.08 |
| ≥ 7days | LR | 0.17 |
|  | RF | 0.16 |
|  | XGB | 0.16 |
|  | LGB | 0.2 |
|  | GB | 0.15 |
|  | MLP | 0.18 |

LR = Logistic Regression. RF = Random Forest. XGB: Extreme Gradient Boosting. GB: Gradient Boosting. MLP: Multi-layer Perceptron. LGB: Light Gradient Boosting.

| **Supplementary Table S5. Patient distribution across hospital length of stay categories in internal and external validation sets** | | |
| --- | --- | --- |
| Hospital Length of Stay | Internal validation set (pts) | External validation set (pts) |
| Day 3 | 42,890 | 59,801 |
| Day 4 | 35,798 | 30,907 |
| Day 5 | 27,013 | 17,719 |
| Day 6 | 12,724 | 13,710 |
| Day ≥ 7 | 11,513 | 47,028 |


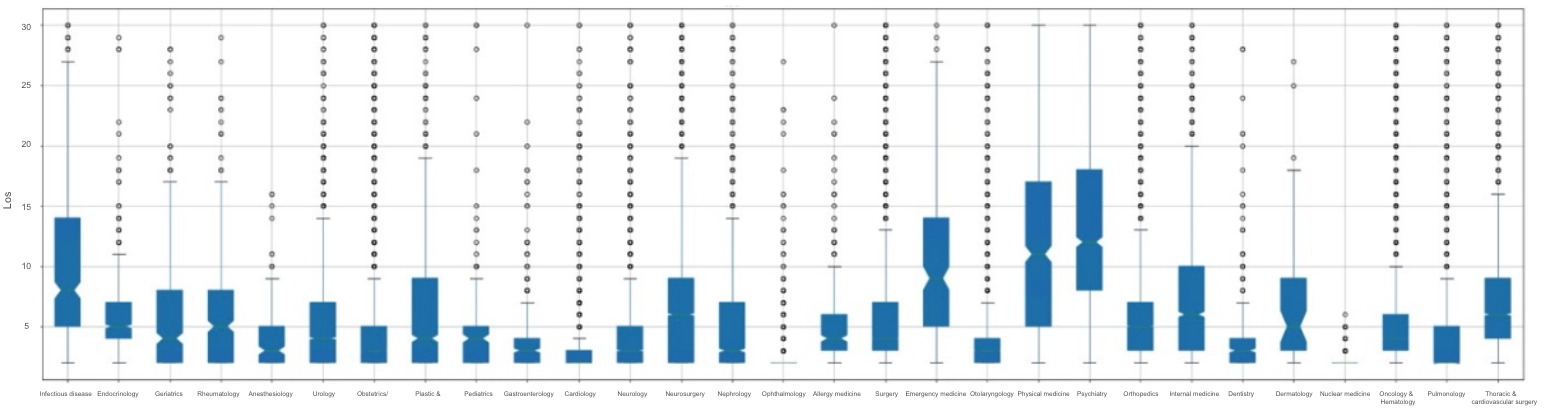


**Supplementary Figure S1.** Distribution of length of stay across admission departments in the internal validation set


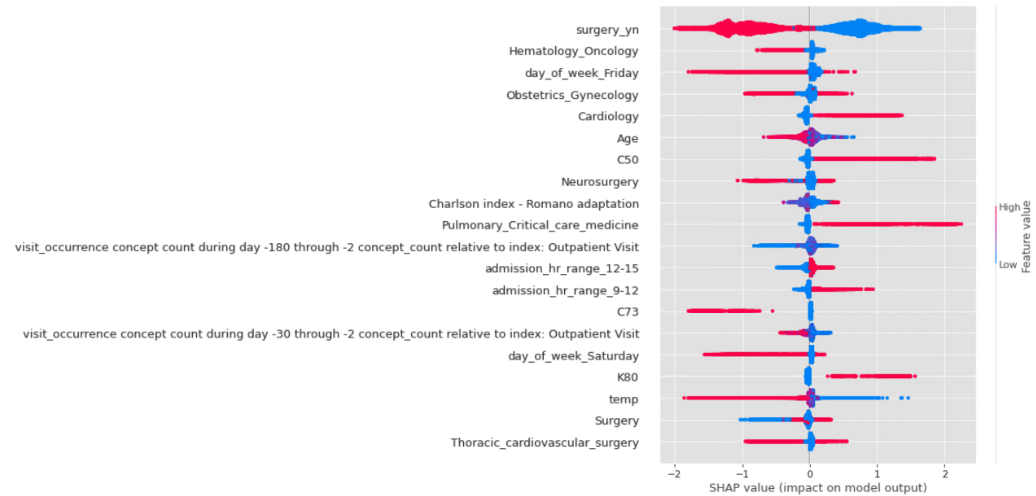

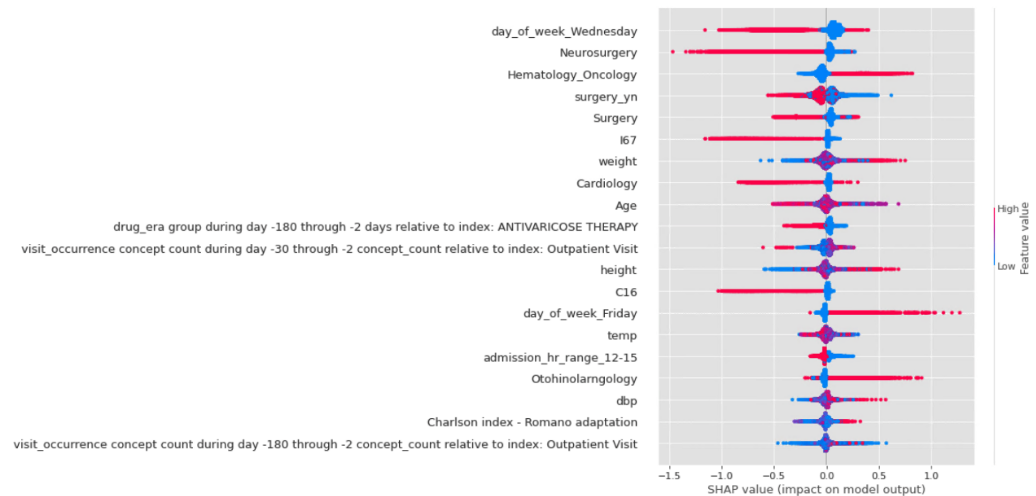

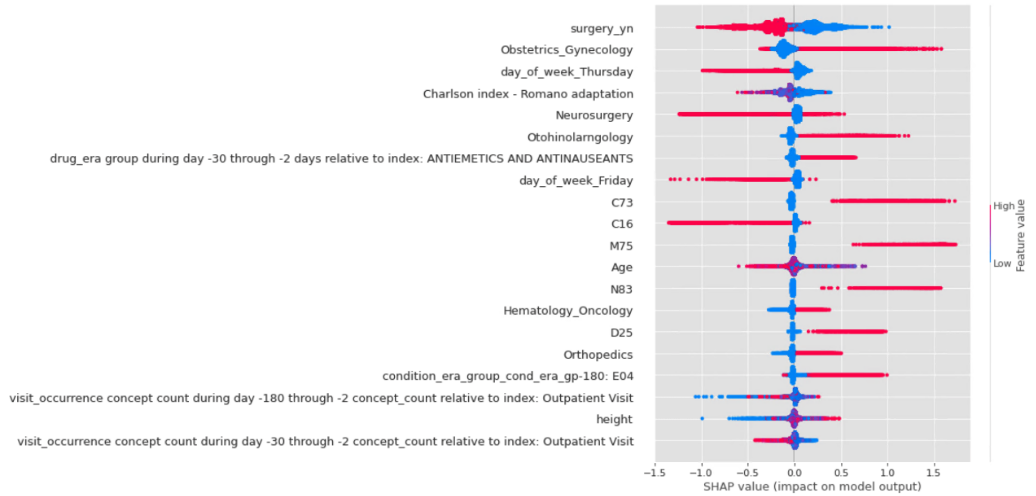


Hospital admission - Wednesday

Neurosurgery

Hematology Oncology

Surgery, Y

Admission to Surgery

I67

Weight

Cardiology

Age

Antivaricose therapy (-180 to -2D)

Surgery, Y

Obstetrics Gynecology

Hospital admission - Thursday

Charlson Index – Romano adaptation

Neurosurgery

Otorhinolaryngology

Antiemetics and Antinauseants (-30 to -2D)

Hospital admission - Friday

C73

C16

Surgery, Y

Hematology Oncology

Hospital admission - Friday

Obstetrics Gynecology

Cardiology

Age

C50

Neurosurgery

Charlson index – Romano adaptation

Pulmonary Critical Care Medicinez

LoS of 4 days

LoS of 5 days

LoS of 3 days


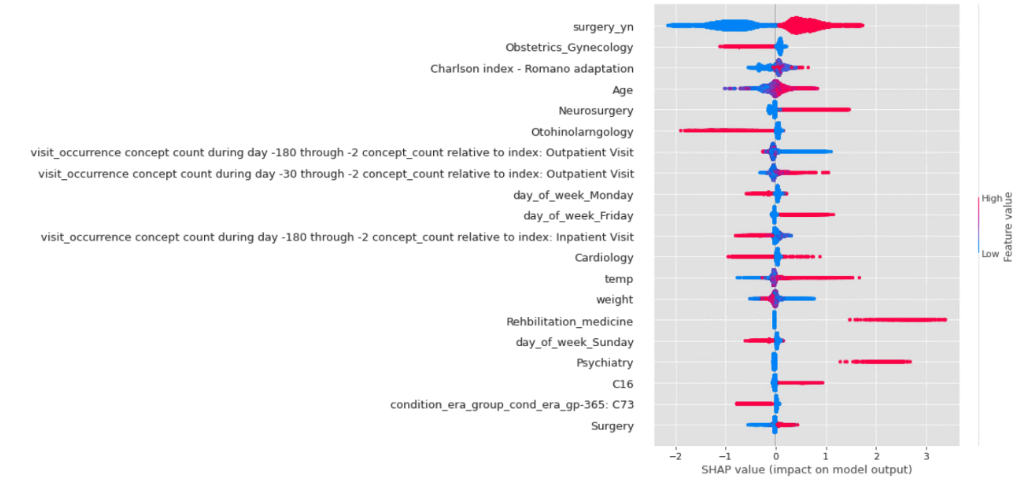


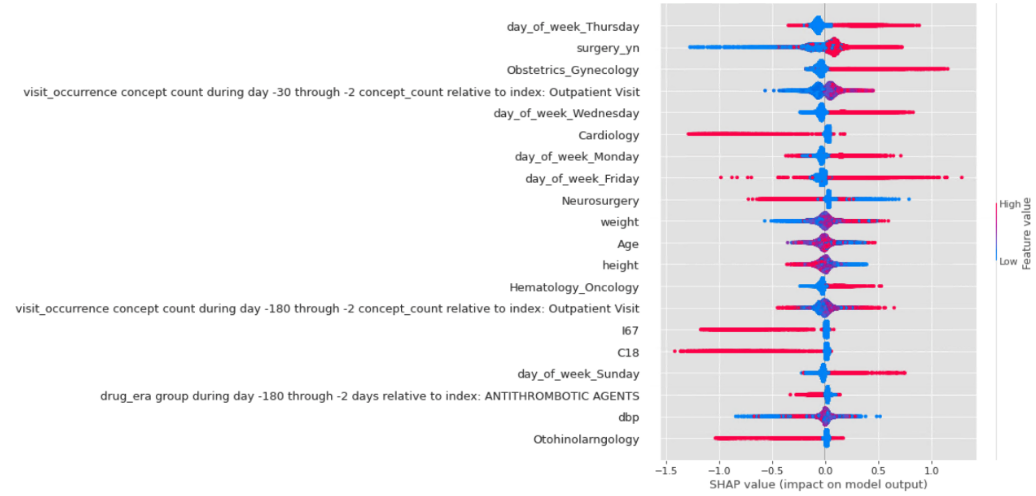


Surgery, Y

Obstetrics Gynecology

Charlson index – Romano adaptation

Age

Neurosurgery

Otorhinolaryngology

No. of outpatient visit (-180 to -2D)

No. of outpatient visit (-30 to -2D)

Hospital admission – Monday

Hospital admission - Friday

Hospital admission-Thursday

Surgery, Y

Obstetrics Gynecology

No. of outpatient visit (-30 to 2D)

Hospital admission – Wednesday

Cardiology

Hospital admission – Monday

Hospital admission – Friday

Neurosurgery

Weight

LoS ≥ 7 days

LoS of 6 days

**Supplementary Figure S2. SHAP analysis for multi-class prediction using the internal validation set**

C50: Malignant neoplasm of breast; C73: Malignant neoplasm of thyroid gland; C16: Malignant neoplasm of stomach; I67: Other cerebrovascular disease; No.of outpatient visits (-30 to 2D): visit occurrence concept count during day -30 through -2 concept count relative to index; No.of outpatient visits (-180 to 2D): visit occurrence concept count during day -180 through -2 concept count relative to index


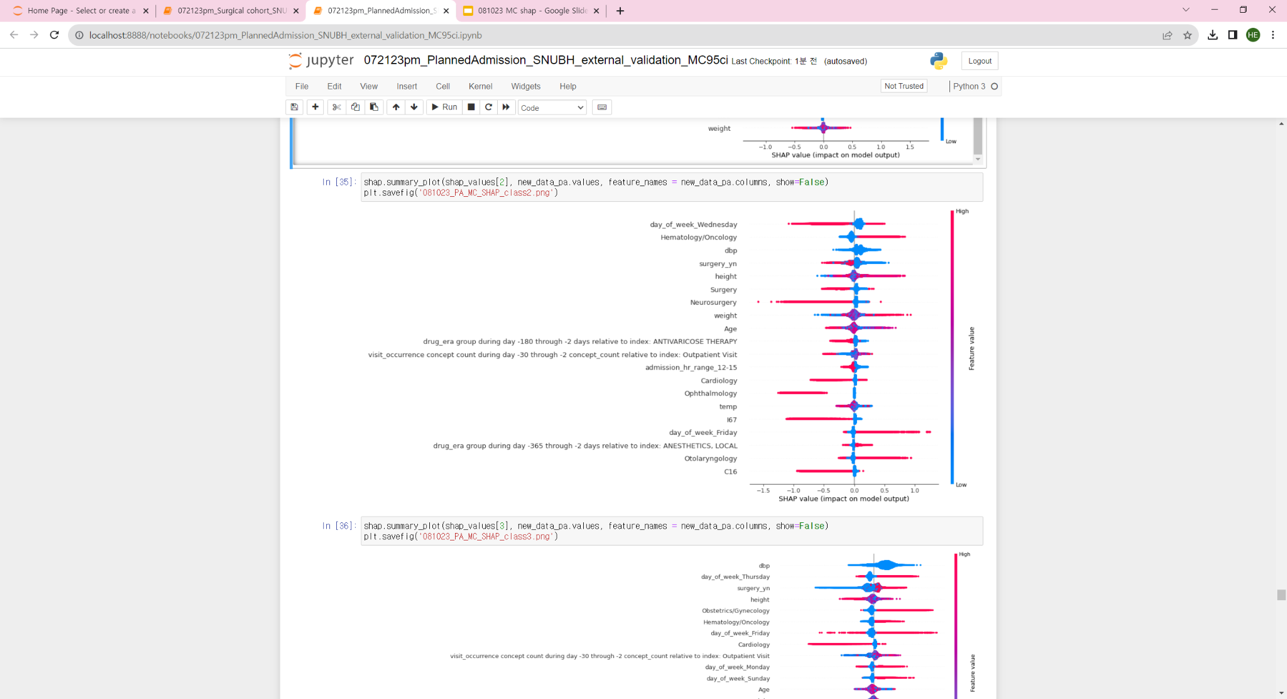

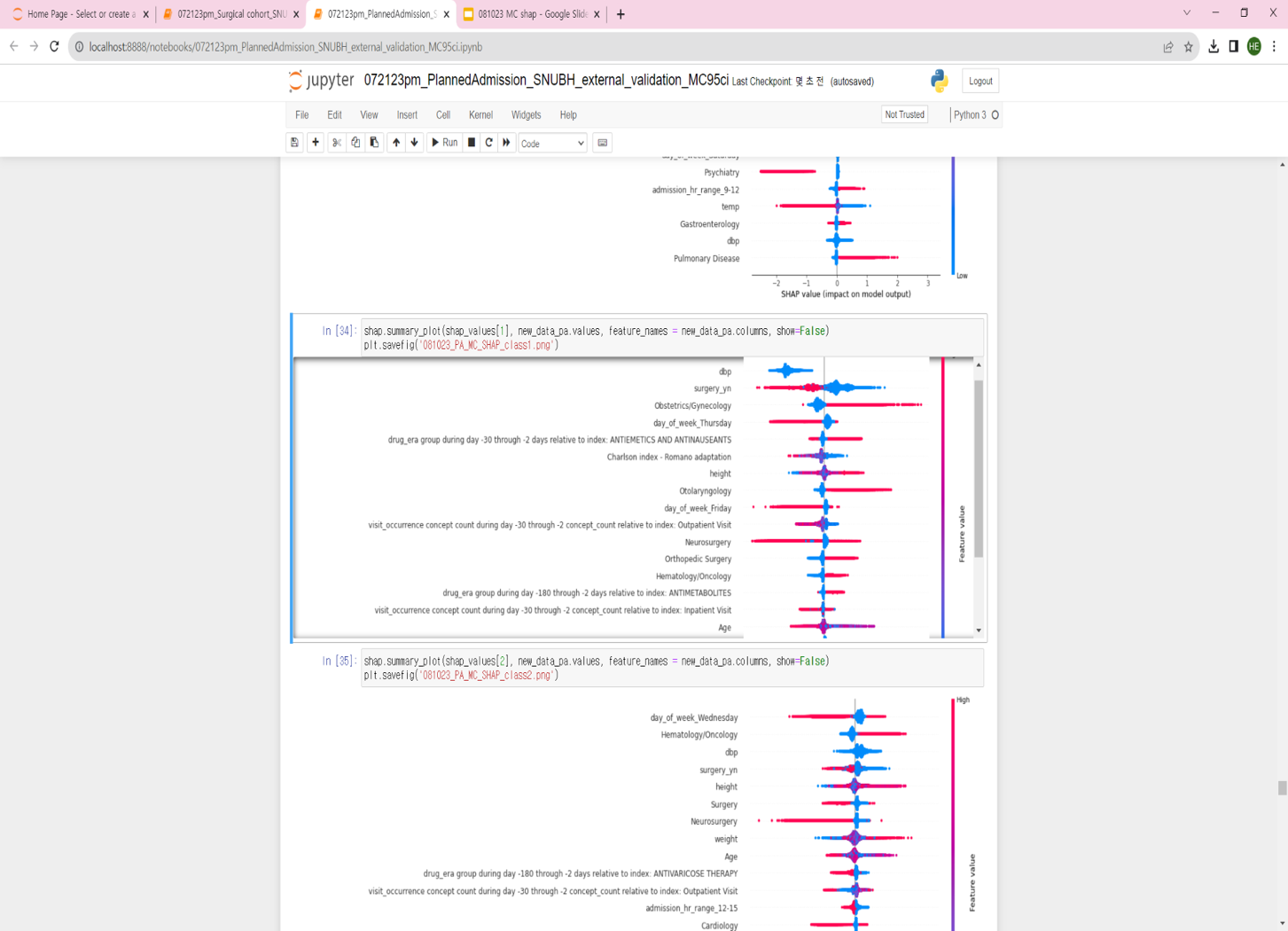

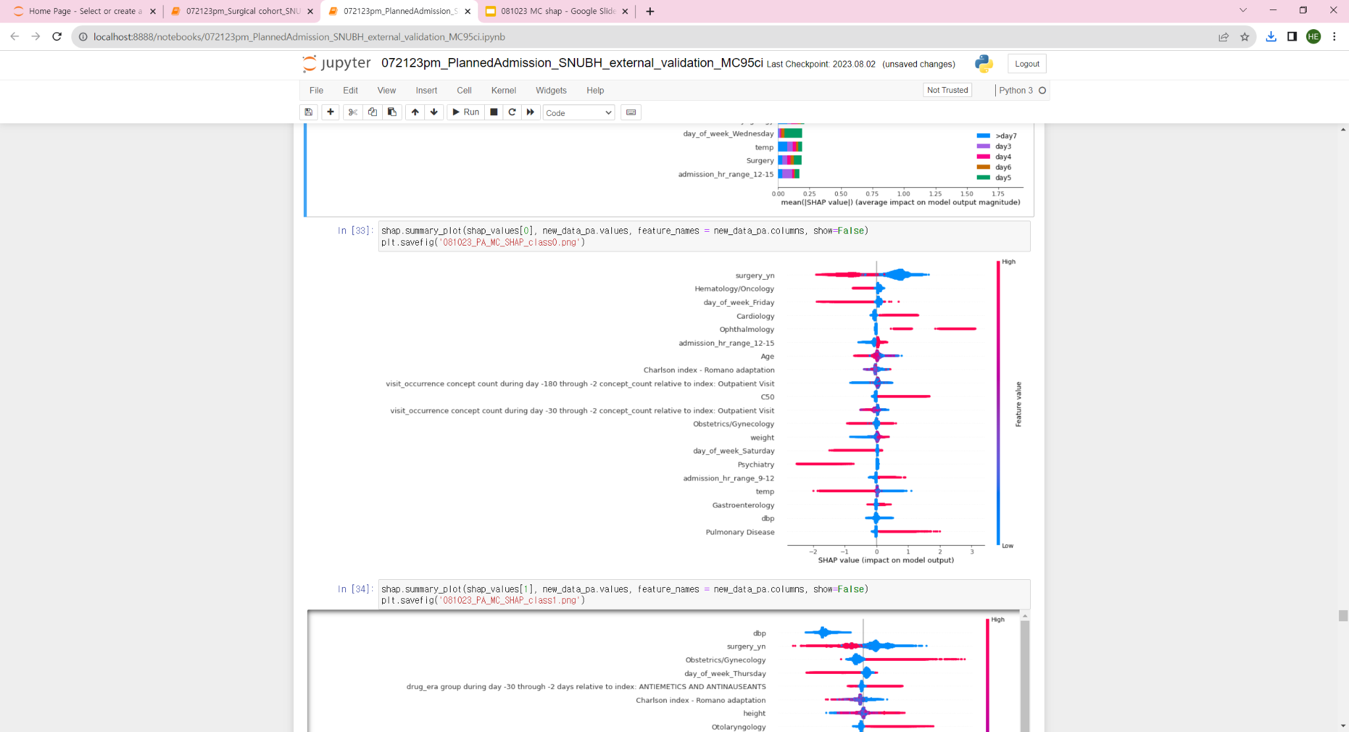


Diastolic blood pressure

Surgery, Y

Obstetrics/Gynecology

Hospital admission – Thursday

Antiemetics and antinauseants (-30 to 2D)

Charlson index – Romano adaptation

Height

Otolaryngology

Hospital admission – Friday

No. of outpatient (-30 to 2D)

Surgery, Y

Hematology/Oncology

Hospital admission – Friday

Cardiology

Ophthalmology

Admission between 12-3PM

Age

Charlson index – Romano adaptation

No. of outpatient visit (-180 to -2D)

C50

Hospital admission – Wednesday

Hematology/Oncology

Diastolic blood pressure

Surgery, Y

Height

Admission to Surgery

Neurosurgery

Weight

Age

Antivaricose therapy (-180 to -2D)

LoS of 3 days

LoS of 4 days

LoS of 5 days


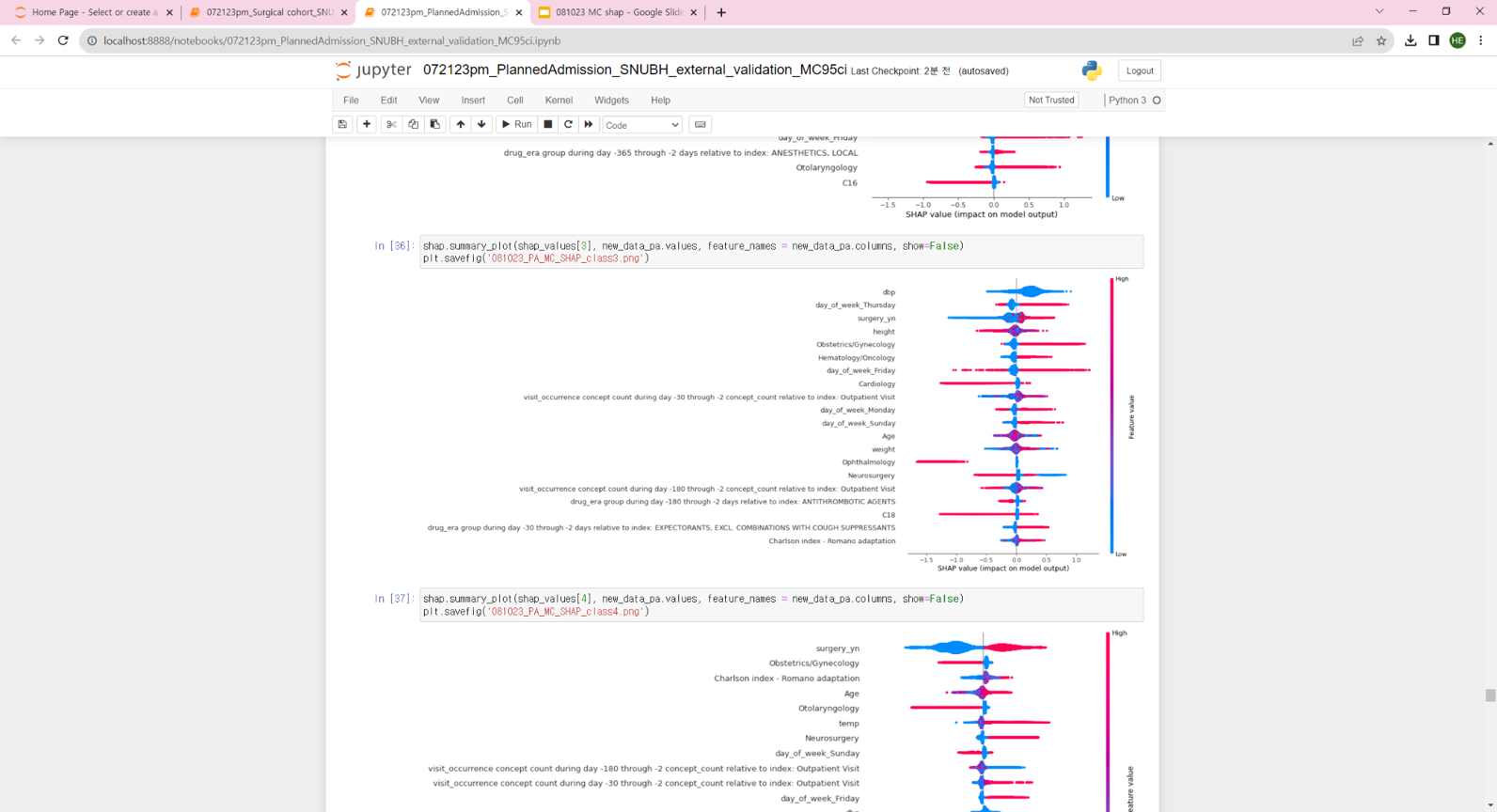

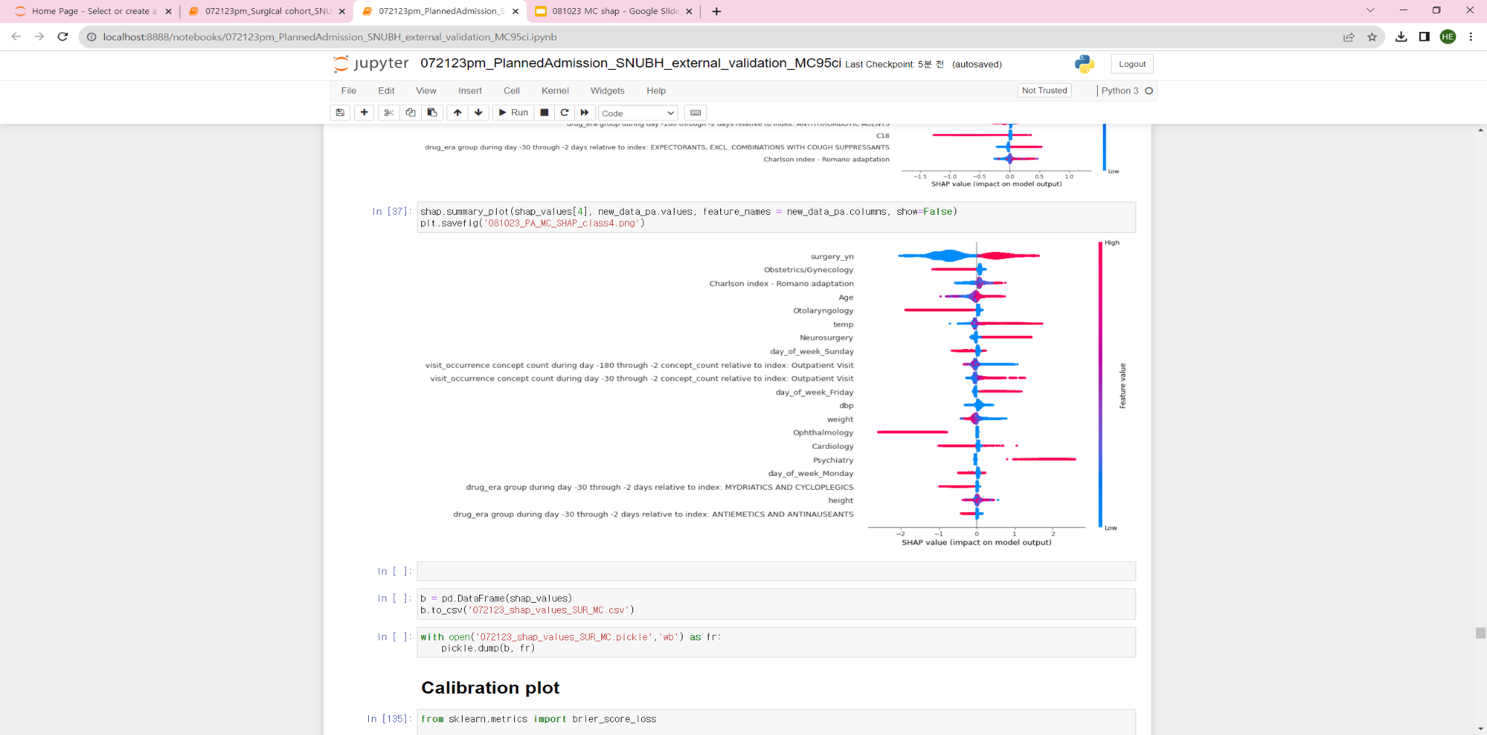


LoS ≥ 7 days

Diastolic blood pressure

Hospital admission – Thursday

Surgery, Y

Height

Obstetrics/Gynecology

Hematology/Oncology

Hospital admission – Friday

Cardiology

No. of outpatient visits (-30 to -2 D)

Hospital admission - Monday

Surgery, Y

Obstetrics/Gynecology

Charlson index – Romano adaptation

Age

Otolaryngology

Body temperature

Neurosurgery

Hospital admission – Sunday

No. of outpatient visit (-180 to -2D)

LoS of 6 days

**Supplementary Figure S3. SHAP analysis for multi-class prediction using the external validation set**

C50: Malignant neoplasm of breast; No.of outpatient visits (-30 to 2D): visit occurrence concept count during day -30 through -2 concept count relative to index; No.of outpatient visits (-180 to 2D): visit occurrence concept count during day -180 through -2 concept count relative to index
